# A plant secretory sequence enhances immunogenicity of electroporated COVID-19 DNA vaccines

**DOI:** 10.3389/fmedt.2025.1597179

**Published:** 2025-07-14

**Authors:** Olivia Costantina Demurtas, Flavia Novelli, Doriana Triggiani, Caterina Merla, Emanuela Pasquali, Silvia Massa, Rosella Franconi, Claudio Pioli

**Affiliations:** ^1^ Energy and Sustainable Economic Development, Division of Biotechnologies (SSPT-BIOTEC), ENEA, Italian National Agency for New Technologies, Rome, Italy; ^2^ Energy and Sustainable Economic Development, Division of Sustainable Agrofood Systems (SSPT-AGROS), ENEA, Italian National Agency for New Technologies, Rome, Italy

**Keywords:** DNA vaccines, plant secretory sequence, electro-gene transfer (EGT), COVID-19, SARS-CoV-2, receptor-binding domain (RBD)

## Abstract

**Introduction:**

As paradigmatically shown by SARS-CoV-2 vaccines, nucleic acids-based vaccines represent powerful tools to rapidly tackle fast emerging pathogens limiting their spread in human populations and/or reducing the health impact in affected patients. Compared with RNA vaccines, DNA vaccines offer higher stability and amenability to fast development due to tailor-made design of several candidates at a time for (pre)clinical settings. However, their scarce immunogenicity represents an important drawback, requiring technological strategies to enhance cellular uptake, protein expression and increase the ability to induce an immune response.

**Methods:**

We investigated the effects of combining a plant secretory signal sequence of the PolyGalacturonase-Inhibiting Protein (PGIP) from *Phaseolus vulgaris* with electro-gene transfer (EGT), a technology that increases DNA delivery, on the immune response induced by different SARS-CoV2 experimental DNA vaccines based on domains and peptides of the spike (S), membrane (M) and nucleocapsid (*N*) proteins.

**Results and discussion:**

All the DNA constructs resulted in protein expression *in vitro* and in the induction of both antibody and CD4 and CD8T cell responses in mice. EGT significantly increased DNA constructs immunogenicity, especially for the induction of antibody response, confirming its potential in DNA vaccination. Remarkably, constructs including the plant secretory signal sequence resulted to be highly expressed and triggered higher antibody and CD4T cell responses, highlighting that the combination of this sequence and EGT can be used to boost the immunogenicity of DNA-vaccine coded proteins, ultimately helping in their design.

## 1 Introduction

The SARS-CoV-2 pandemic, tackled through quarantine protocols and emergency vaccination programs, highlighted the importance of rapid development of new vaccines when emergent pathogens arise. Two main nucleic acid-based platforms were used, namely mRNA and non-replicative adenoviral vector-based vaccines.

Controlling future outbreaks at global level not only requires a fast way to develop vaccines, but also a technology that ensures a successful triggering of a sustained, non-deleterious, immunological response. DNA vaccination can contribute to address both issues (1). However, integrity of DNA and route of administration of genetic vaccine are crucial to generate the desired immunity. One of the drawbacks of DNA-based vaccines is the limited ability of “naked” DNA to cross the plasma membrane.

DNA injection followed by high-voltage electric pulses or combinations of high-voltage and low-voltage pulses results in the delivery of DNA constructs by cell/nuclear membrane electroporation (EP) leading to high expression of the encoded proteins. This Electro-gene transfer (EGT) approach results in localized inflammatory responses that help immune activation. EGT has been successfully applied to a wide range of tissues, including muscle, skin, heart, liver, lung, and vasculature. Unlike viral vectors, EP does not require the use of viral components, reducing the risk of anti-vector immune responses and improving the safety profile (2). These advantages have brought the EGT to the clinical stage of research for a wide range of applications, including infections and cancer (1, 3–10).

A second challenge for DNA vaccines is the relatively lower antibody response compared to other vaccine platforms. Specifically, for SARS-CoV-2 DNA vaccines, the reported responses were approximately 2-log lower than those observed for mRNA vaccines and about 1-log lower than those induced by adenoviral vaccines (11). Common approaches to stimulate immunity of DNA vaccines include the use of genetic adjuvants (i.e., CpG motifs, cytokines, chemokines, and heat shock proteins) (12, 13). Signal sequences have been also shown to effectively improve the immunogenicity of genetic vaccines by promoting efficient antigen processing and presentation (14, 15). Signal sequences, typically located at the N-terminus of proteins, direct nascent polypeptides to the endoplasmic reticulum (ER) for secretion or membrane localization. This targeting facilitates proper folding, post-translational modifications, and subsequent antigen presentation. Fusion of a secretory signal peptide sequence can lead to increased humoral and CD8^+^ T cell responses, as shown for an anti-hepatitis B surface antigen DNA vaccine (16). Similarly, research on mRNA SARS-CoV-2 vaccines has shown that optimizing signal sequences can enhance antigen expression, leading to more robust humoral and cellular immune responses (17). We previously demonstrated that a signal sequence derived from a plant, specifically the secretory sequence of the PolyGalacturonase-Inhibiting Protein (PGIP) of *Phaseolus vulgaris,* can change the sorting of heterologous proteins in mammalian cells, eliciting a stronger immune response to a DNA vaccine (18).

Another issue, in order to tackle potential re-emerging pandemics, is to obtain protection against all the coronaviruses (pan-reactive vaccines) or a subset of coronaviruses (broadly protective vaccines) (19). The licensed vaccines against the SARS-CoV-2, with the exceptions of the inactivated virus that present all the viral antigens, are based on the receptor binding domain (RBD) of the SARS-CoV-2 spike (S) protein. The RBD was the first and main selected viral protein target to induce neutralizing antibodies, due to its surface localization and role in virus cell entry, as also previously shown for other human coronaviruses (20). However, the spike protein is the most prone to mutation SARS-CoV-2 antigen, leading to different virus variants and sub-variants able to evade the immune response elicited by the original RBD spike protein (21). Therefore, the next generation vaccines are expected to include other highly conserved structural and non-structural SARS-CoV-2 proteins, able to induce protection by cross-reactive CD4 and CD8T cells (19, 22, 23).

In this work we studied the effects of the secretory sequence (ss) from PGIP in combination with EGT on the ability of anti-SARS-CoV-2 DNA vaccines to induce antibody and T cell immune responses.

## 2 Materials and methods

### 2.1 DNA constructs

Chimeric constructs (see paragraph 3.1) were designed by our group, synthesized by GenScript Co (USA), with codons optimized for insect cells (*Spodoptera frugiperda*), and cloned into the pVAX1 vector (Invitrogen, Cat No. V26020). Codon-optimized constructs were designed considering codon usage bias, GC content, decrease of negative *cis*-acting sites and repeat sequences, and restriction sites that may interfere with cloning. To improve translation of the mRNA, a Kozak consensus sequence was inserted into the synthetic genes. One milligram of each plasmid, endotoxin-free, diluted in bi-distilled water, was produced. The plasmids were also re-amplified in XL1 blue *E. coli* cells and extracted with the Maxi-prep endotoxin-free kit (Sigma NAO400). Analysis of 3D structure and immunogenic peptides was performed by Protean 3D (Lasergene, DNA star) software. Alignment of protein sequences was performed with Clustal Omega.

### 2.2 *in vitro* transfection

HEK-293 cells (ATCC CRL-1573, USA) were cultured as a monolayer in Dulbecco's modified Eagle's medium containing 4.5 g/L glucose, supplemented with 10% fetal bovin serum (FBS), 1 mM sodium pyruvate, 100 units/ml of penicillin/streptomycin (all Corning, USA) and maintained at 37°C with 5% of CO_2_ and relative humidity of 95%. For seeding, cell lines were harvested at confluence with 0.25% Trypsin/EDTA. One day prior to transfection, 200.000 viable cells/ml were seeded on sterilized coverslips on 24-well plate resulting in ∼70% confluency on the day of transfection. Cells were transfected with vectors using the transfection reagent TransIT®-293 Reagent (Mirus bio, #MIR 2704) according to the manufacturer's protocol. TransIT®-293: DNA construct complexes, obtained by incubating 500 ng of plasmid DNA and 1.5 µl of TransIT®-293 reagent in Opti-MEM® medium (Gibco™, #31985070) for 15–30 min at room temperature, were added dropwise to cells grown on glass coverslip. Optimal TransIT®-293: DNA ratio was assessed in preliminary experiments. After 48 h, the expression of the constructs was assessed by immunofluorescence*.*

### 2.3 Immunofluorescence microscopy

Transfected HEK-293 cells adhered to glass coverslips, washed with phosphate buffered saline (PBS), were fixed and permeabilized with ice-cold 1:1 acetone/methanol for 5 min. After washing with PBS, non-specific binding sites were blocked by incubating the coverslips with 3% FBS in PBS for 90 min at 37°C. After removal of the blocking solution, SARS-CoV-2 Spike Protein Receptor Binding domain (RBD) Polyclonal Antibody (1:750 diluted; Invitrogen PA5-114451) or SARS/SARS-CoV-2 Nucleocapsid (*N*) Polyclonal Antibody (1:750 diluted; Invitrogen PA5-119601) in 1% FBS/PBS was added, followed by 1.5 h incubation at room temperature. After washes, cells on coverslips were incubated for 1 h at room temperature with Alexa Fluor488-conjugated goat anti-rabbit IgG (1:750 diluted, Invitrogen A-11034) and with 0.1 μg/ml of DAPI (4′,6-diamidino-2-phenylindole) for counter staining in 1% FBS/PBS. Images were captured using an inverted light confocal microscope (Zeiss, Axio Observer, Jena, Germany) with a 10X objective.

### 2.4 Animals, treatments and sample collection

The study was carried out according to the European Community Council Directive 2010/63/EU. The experimental protocol was approved by the Body for the Protection of Animals (OPBA) of ENEA and authorized by the Italian Ministry of Health (127/2022-PR). Six-eight weeks old female BALB/c mice were purchased by Charles River Laboratories Italia (028BALB/C). Mice were divided into 6 groups (5–13 mice/group) according to the type of injected constructs and/or the application of the electroporation protocol. Control mice were injected with the vehicle (water). Mice were anesthetized by isoflurane 2%, injected intra-muscularly (i.m.) with one of the four constructs (25 µg DNA in 25 µl bi-distilled water/mouse) into the right and left tibialis anterior or with vehicle (water) and treated or not with electroporation twice, 3 weeks apart (day 0 and 21). Two weeks after priming (day 14) and after boost (day 35), sera were collected and analyzed by enzyme-linked immunosorbent assay (ELISA). On day 35 the spleens were also collected for T cell analyses by flow cytometry.

### 2.5 Electro-gene transfer (EGT)

EGT was carried out immediately after DNA construct (or vehicle) injection on both limbs using two brass flat electrodes (inter-electrode distance 4 mm) applying 8 pulses lasting 20 ms each (at full width at half maximum) for an applied electric field of 175 V/cm at a repetition frequency of 1 Hz using a veterinary generator (Electro Cell B15 from Leroy Biotech, France). All the delivered pulses were recorded by the generator for both the voltage applied to the electrode and the delivered current to verify the effectiveness of each treatment. The measured current by the instrument is essentially composed of two components: (1) current flowing through the echography gel across the electrodes and (2) the current flowing through the mouse leg. This latter current component was estimated using a simple model evaluating the ratio of the applied voltage across the electrode and the resistance of the mouse leg. The global resistance is obtained as the ratio between the electrode length (l equal to 1 cm) and the product of half the radius squared, multiplied by pi, and the leg conductivity (R = l/[(r/2)^2^ ∗ π ∗ s] where R is the resistance, l is the electrode length, r is the radius of the mouse leg measured at the point of its maximum width and s is the tissue conductivity). The conductivity of the leg is an average of the conductivity of the main tissues composing the animal leg, such as muscle and bone, estimated to be equal to 0.14 S/m. Half of the mouse leg radius (r/2) was considered in this estimation as a reasonable approximation of the real leg section, as it varies along the electrode length. Mouse leg radius was extracted from the leg circumference measured by a caliper for each electroporated mouse at the point of the maximum leg width. The radius values used for the current assessment ranged from 24–35 mm.

### 2.6 ELISA

Serum samples were tested by ELISA to detect anti-RBD and anti-N IgG. Briefly, blood samples were collected into tubes without additives, kept at room temperature for 30 min and, after clotting, centrifuged at 3,000 rpm and 4°C for 10 min. 96-well EIA/RIA Polystyrene High Bind Microplates (Corning 3,590) were coated with 10 µg/ml of RBD or N protein in PBS and incubated overnight at 4°C. PBS-Bovine Serum Albumin (BSA) 1% was used to block unoccupied binding sites. Then, serially diluted serum samples were added to plates and incubated at 37°C for 1 h. Washes were performed with PBS-Tween 20 (0.05%). Detection was performed using a Peroxidase AffiniPure Goat Anti-Mouse IgG, F(ab′)₂ fragment specific (Jackson ImmunoResearch, JACK-115-035-006; diluted 1:5,000) followed by the incubation with the substrate solution (2,2′-Azino-bis(3-ethylbenzothiazoline-6-sulfonic acid) (SIGMA A-3219). Absorbance was read at 450 nm. Preliminary assays were run to setup optimal conditions for ELISA. The area under curve (AUC) of the O.D. vs. serum dilution curves was calculated using the GraphPad Prism 8 software for each mouse sample. Results are shown as individual AUC values, means ± S.E. of AUC values for each group, and as frequency of positive mice/group. Individual samples were defined as positive if their AUC values exceeded the mean AUC value of not immunized mice (vehicle control group) by more than 2 SD.

### 2.7 T cell antigen stimulation

On day 35, two weeks after the boost, spleen cells were collected and dissociated to single cell suspensions. After removal of red blood cells, leukocytes were cultured with medium or stimulated with either RBD-S2′-M-N (31.13 kDa) or RBD (27.2 kDa), or N protein (10 µg/ml each) for 5 days. RBD-S2′-M-N and RBD proteins were purchased from GenScript Co. by customized protein production in *E. coli* of synthetic sequences with codon optimized for bacterial expression. The amino acid sequences are identical to those codified by the respective constructs used for DNA vaccination. The N protein was purchased from Genscript (Z03488-100). During the last 5 h of culture, cytokine production was boosted by adding again the respective proteins, in the presence of monensin and brefeldin A (00-49-80, Thermofisher). Parallel cultures received medium (negative controls) or Phorbol 12-myristate 13-acetate (PMA)/ionomycin (positive controls). At the end of the re-stimulation period, cells were collected and analyzed by flow cytometry.

### 2.8 Flow cytometry analysis

Spleen single cell suspensions were stained with the Fixable Viability Dye (FVD)-eFluor450 (65-0863-14, Thermofisher) to assess viability. Then, cells were incubated with an anti-CD16/32 mAb (Fc block, 553142, BD Biosciences) to prevent binding of antibodies to FcRs and stained with CD3-FITC (MA1-10187), CD8-eFluor506 (69-0081-82), anti-IFN-ɣ-PE-eFluor610 (61-7311-82), and anti-TNF-α-PE-Cy7 (25-7321-82) Abs (all from Thermofisher). For intracellular staining, cells were fixed and permeabilized using the Foxp3/Transcription Factor Staining Buffer Set (00-5523-00, eBioscience). Optimal concentrations of the Abs were assessed by titration and staining index values in preliminary experiments. 10^5^–2 × 10^5^ total events/sample were collected using a 4 lasers CytoFLEX S flow cytometer (Beckmann Coulter). Cells cultured with medium, and cells stimulated with PMA and ionomycin were used as negative and positive controls, respectively. Data were analyzed using the FCS Express software (*de novo* Software). FMO (fluorescence minus one) samples were used to set quadrants separating negative/positive cells for cytokine expression. The hierarchical gating strategy is described in the supplementary procedures.

### 2.9 Statistical analysis

Mice were individually analyzed for all the parameters investigated. One-way ANOVA followed by Tukey-Kramer *post hoc* test analyses were performed for comparison between groups. Data are shown as means ± standard error of mean (S.E.). The number of mice for each specific group is indicated in figure legends. *p* values <0.05 were considered statistically significant.

## 3 Results

### 3.1 Design of DNA constructs

We designed four genetic constructs based on the receptor binding domain (RBD) of the spike (S) protein combined with other sequences of SARS-CoV-2 (GenBank: MN908947.3) structural genes (S, nucleocapsid or N and membrane or M). To study the effect of plant secretory sequence on DNA-based construct immunogenicity, we included or not the secretory sequence (ss) of the PolyGalacturonase-inhibiting protein of *Phaseolus vulgaris* (PGIPss) (18).

**Figure 1:**
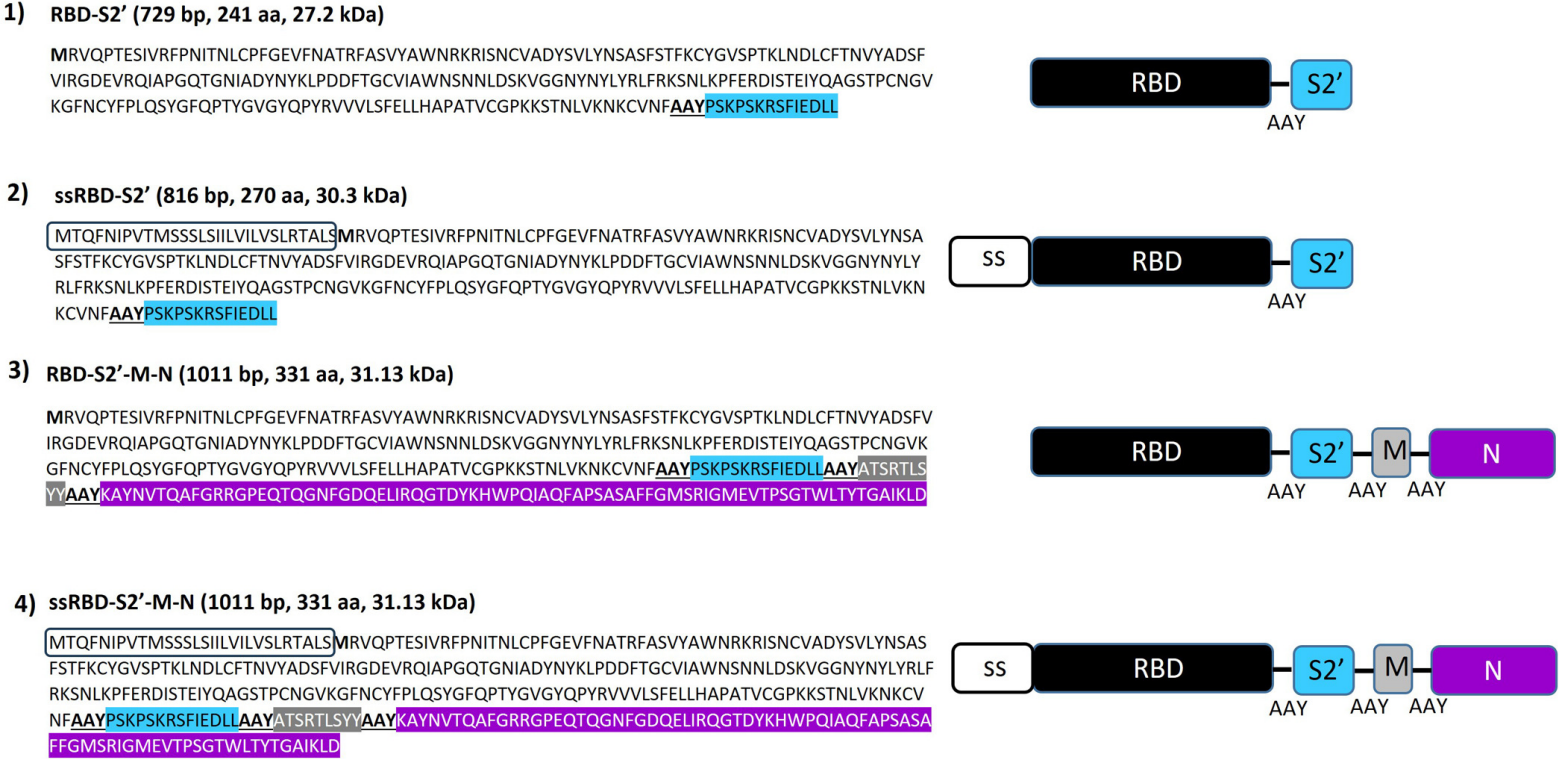
Genetic multi-epitope constructs used for mice vaccination. Four genetic constructs encoding for SARS-CoV2 chimeric proteins/epitopes were designed and used in this study: (1) the receptor binding domain (RBD, aa 319–541) of the S protein fused to the S2′ epitope, aa 815–818, (RBD-S2′); (2) the RBD-S2′ construct N-terminally-fused to the secretory sequence (ss) of the PolyGalacturonase-inhibiting protein (PGIP) of *Phaseolus vulgaris* (ssRBD-S2′); (3) the RBD-S2′ construct fused to the membrane (M, aa 171–179) and nucleocapsid (N, aa 266–340) protein epitopes,; (4) ssRBD-S2'-M-N, that consists of construct number 3, N-terminally-fused to the PGIPss. The different peptides were spaced using the 3 amino acids AAY as linker. The RBD of the SARS-CoV-2 S protein is reported in black letters after the first inserted methionine in bold, the S2′ domain of the S protein is in light blue, the PGIP secretory sequence (ss) is included in the rectangle, the M and N epitopes are highlighted in grey and violet, respectively. The 3 aa spacer AAY is bold and underlined.

All the constructs included the RBD sequence (aa 319–541 of the S protein, GenBank: BCN86353.1). We chose the sequence that harbors the 3 mutations (K417 > N; E484 > K; N501 > Y) found on the B.1.351 variant strain (24), followed by a 3 amino acid spacer AAY (25) and by a peptide comprising the S protein KRSF domain (aa 815–818 of the S protein) that corresponds to the S2′ cleavage site (furin-like) (Supplementary Figure S1), that is cleaved between the amino acids R and S (26), and it is essential for viral entry (27). The rationale behind this choice consists of the possibility to stimulate the production of antibodies that prevent the entrance of the virus into the cells, after its binding to the ACE2 receptors. Since the S2′ cleavage site consists of only 4 aa, to gain its visibility by the immune systems, we selected a 14 aa peptide comprising this site including 5 aa upstream and a portion of the internal fusion peptide (IFP) downstream (Figure 1).

The four realized constructs (Figure 1 and Supplementary Figure S2), are:

(1) RBD-S2′, that is the vaccine basic element selected in this study;
(2) ssRBD-S2′, that consists of construct number 1, N-terminally-fused to PGIPss. We had previously used this plant signal sequence to drive other pathogen-related antigens into the human secretory pathway, demonstrating that it is able to modulate the sorting of heterologous proteins in mammalian cells and to increase humoral response in the context of DNA-based vaccines (18).
(3) RBD-S2′-M-N, that consists of construct number 1, C-terminally fused to selected epitopes of the M and N proteins. The different peptides were spaced using the 3 amino acids AAY linker (25).
(4) ssRBD-S2'-M-N, that consists of construct number 3, N-terminally-fused to the PGIPss.

For the M protein (Gene bank YP_009724393.1) we selected the immunogenic CTL epitope ATSRTLSYY (aa 171–179) (28–30), that is the unique probable non allergen epitope reported (28) (Supplementary Figure S3A).

For the N protein (GenBank: MN908947.3), we selected a long peptide (Supplementary Figure S3B) containing 3 immunogenic epitopes: KAYNVTQAF (aa 266–274) (probable non allergen peptide selected from (30), ELIRQGTDY (aa 290–2298) (probable non allergen peptide selected from (30), and GMEVTPSGTWLTYTGAIKLD (aa 321–2340) [CD8T cell epitope, (31)].

Before proceeding with the realization of the constructs, we studied the structure of the chimeric proteins by using Protean 3D software (Lasergene, DNA star). Since the PGIPss should be removed in the mature proteins (18), we modelled the constructs 1 and 3, which lack the ss, and we observed that the proteins have few disordered regions, good surface probability for the epitopes of interest (in Supplementary Figure S4 we show a picture of 3D models obtained, in which the S2′, M and part of the N epitopes appear exposed on the surface), and B and T epitopes are present along all the sequence (data not shown).

The chimeric sequences were codon-optimized for the expression in insect cells (*Spodoptera frugiperda*) since we previously demonstrated that the use of this codon usage in human cells lead to higher yields of a recombinant protein (32). Since it is known that GC content and codons abundance are essential factors in to obtain amplification of mRNA transcription rate (33, 34), we optimized the sequences considering the GC content, the decrease of negative *cis*-acting sites and repeat sequences, obtaining the following Codon Adaptation Index (CAI) values for the expression in mammalian cells: RBD-S2′: 0.91, ssRBD-S2′: 0.92, RBD-S2′-M-N: 0.90, ssRBD-S2′-M-N: 0.90.

### 3.2 Expression of DNA constructs in human cells

To validate the four engineered genetic constructs, transient transfections were performed on HEK-293 cells and, after 48 h, the expression of chimeric proteins was determined by immunofluorescence, using polyclonal anti-RBD and anti-N antibodies followed by Alexa Fluor488-conjugated secondary antibody.

**Figure 2:**
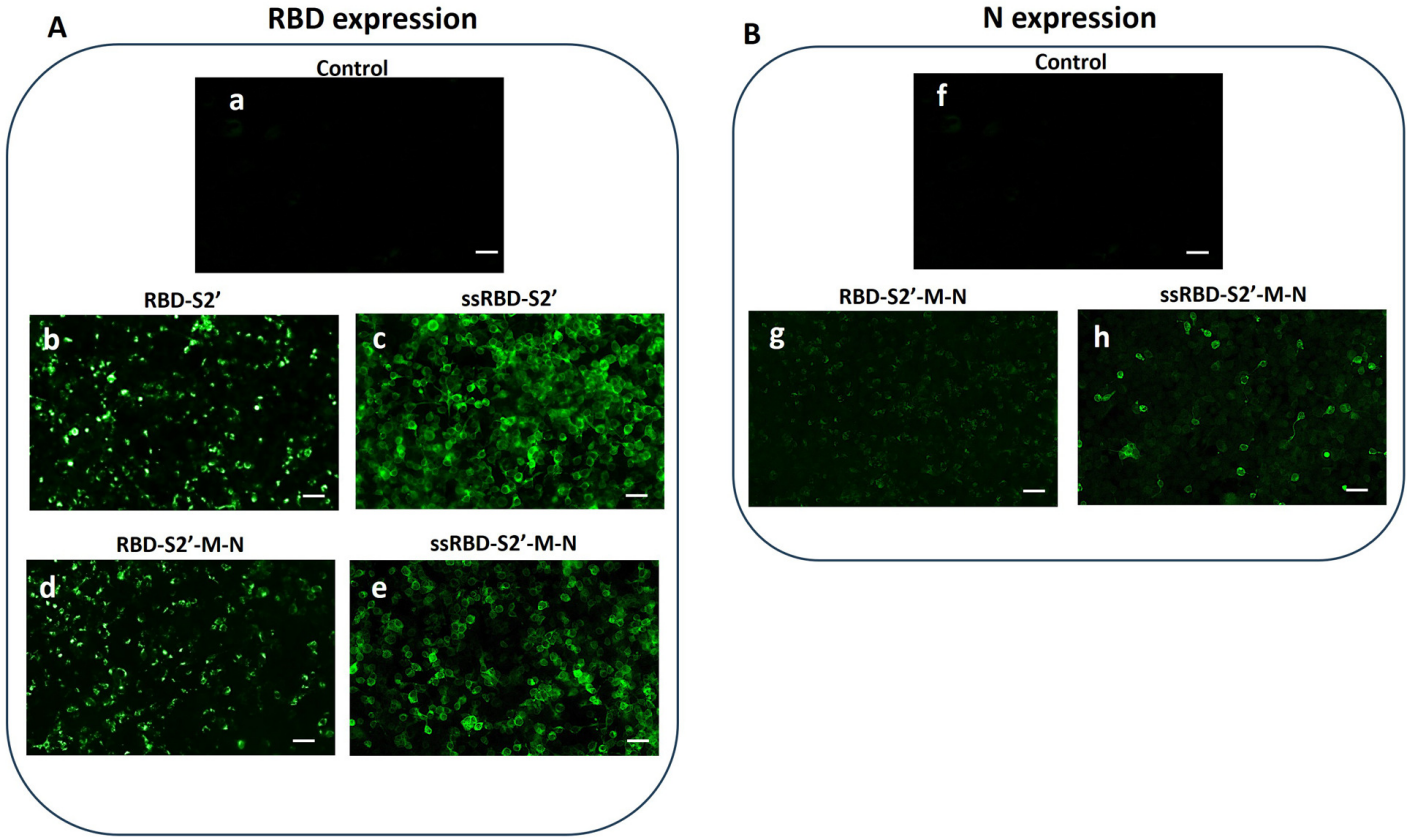
Recombinant multi-epitope proteins are expressed in HEK-293 cells. Immunofluorescence of HEK-293 cells performed 48 h post transfection was recorded by confocal microscopy. **(A)** RBD expression was detected using an anti-RBD polyclonal antibody (see paragraph 2.3 for details) in cells transfected with the RBD-S2 (b), ssRBD-S2′ (c), RBD-S2′-M-N (d) or ssRBD-S2′-M-N (e) constructs. Control: untransfected cells (a); **(B)** N expression was detected using an anti-N polyclonal antibody (see paragraph 2.3 for details) in cells transfected with RBD-S2′-M-N (g) or ssRBD-S2′-M-N (h) constructs. Control: untransfected cells (f); White bar represents 25 µm length.

As shown in Figure 2A, all the DNA constructs allowed the expression of the RBD, albeit at varying rates (panels b-e). Notably, the addition of the PGIPss into the constructs resulted in enhanced RBD expression. Specifically, the immunofluorescence intensity was higher in cells transfected with the ssRBD-S2′ construct (panel c) compared to the RBD-S2′ construct (panel b), and in cells transfected with the ssRBD-S2′-M-N (panel e) compared to the RBD-S2′-M-N (panel d), suggesting that the presence of the PGIPss enhances the expression of the recombinant proteins. The expression of ssRBD-S2′ (panel c) was higher than that of the ssRBD-S2′-M-N (panel e).

Cells transfected with the constructs encoding for RBD-S2′-M-N (with or without the ss) were also analyzed for the presence of N protein (Figure 2B), showing a detectable N protein expression, indicating that the multi-epitopes chimeric proteins are correctly folded, and epitopes displayed. In addition, as previously observed for RBD, the immunofluorescence intensity corresponding to N expression was higher in cells transfected with the construct containing the PGIPss (panel h vs. panel g), confirming that this sequence boosts the expression of the fused proteins.

### 3.3 Mice vaccination by intramuscular DNA injection and electro-gene transfer (EGT)

**Figure 3:**
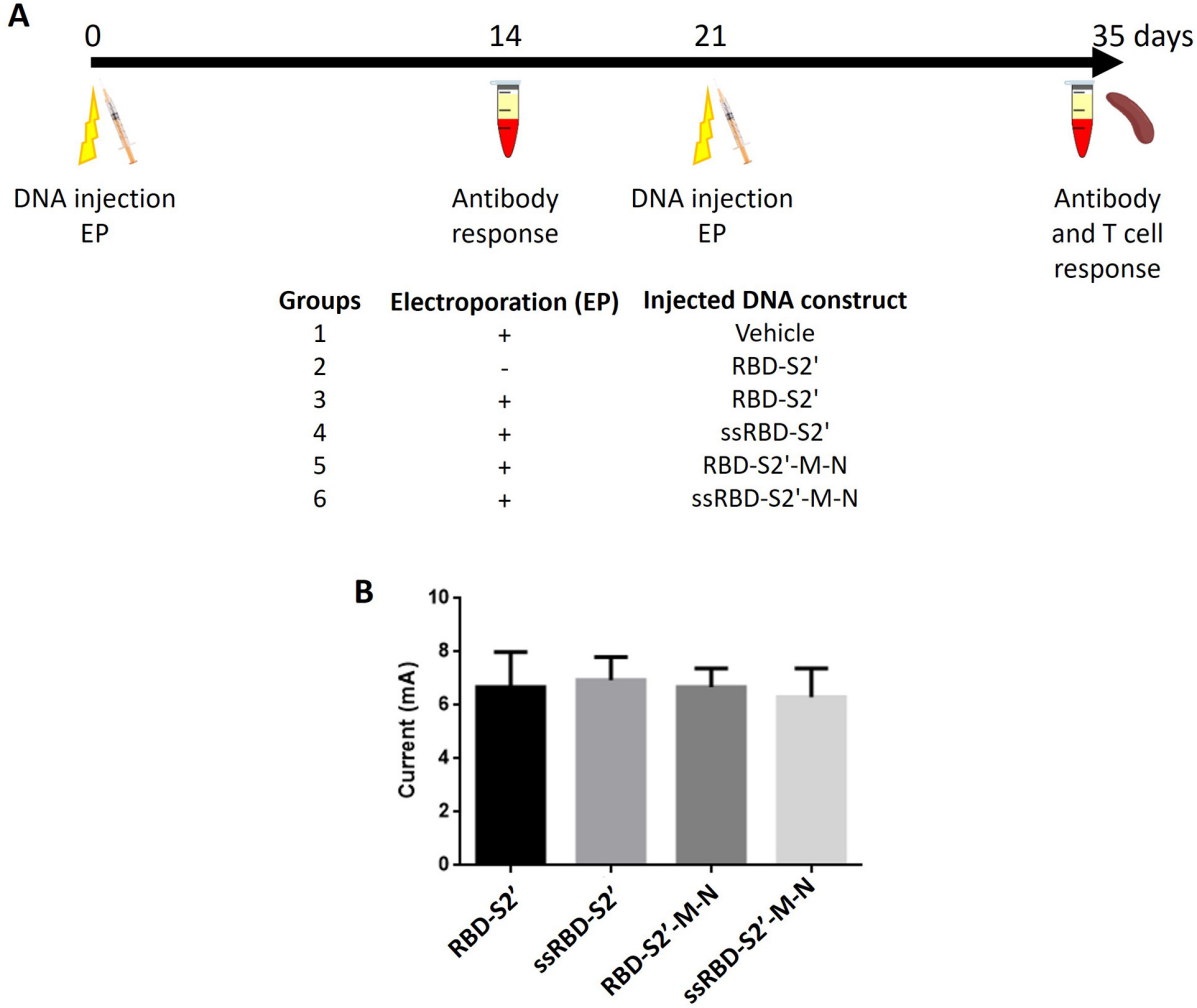
Schematic representation of vaccination protocol and computed current on vaccinated mice subjected to EGT. **(A)** Six to thirteen, six-eight weeks old, female BALB/c mice per group were injected i.m. (both right and left tibialis anterior) with the indicated DNA construct at day 0 and 21. Immediately after injection, mice were treated (groups 1, 3–6) or not (group 2) with electroporation (EP). Two weeks after priming (day 14) and after boost (day 35), sera were collected and analyzed by ELISA. On day 35 also the spleens were collected for T cell analyses by flow cytometry. As control we also included a group of mice vaccinated i.m. without EP with the construct RBD-S2′ (group 2). **(B)** Computed currents in milliampere (mA) were calculated on 5 to 8 vaccinated mice per group considering both prime and boost EGT experiments on both animal legs (average values + standard deviation of the two experiments are shown). Mice/group: 6 for RBD-S2′, 5 for ssRBD-S2′, 5 for RBD-S2′-M-N, 8 for ssRBD-S2′-M-N. Leg diameters have been measured by a caliper as reported in Materials and Methods (paragraph 2.5). No statistically significant differences were found between groups (*p* < 0.05, ANOVA followed by Tukey-Kramer test).

DNA vaccines often induce sub-optimal immune responses, displaying low/scarce immunogenicity and requiring high doses and/or several boosting challenges to obtain optimal protection. In our study, after i.m. injection, animals were subjected to EGT as it favors cell transfection and generates limited local tissue damages, resulting in the induction of an inflammatory response (1, 4, 6–8). BALB/c mice were injected i.m. with DNA constructs and subjected to EGT twice, 3 weeks apart (day 0 and 21). As control, a group of mice was vaccinated with vehicle (water) (Figure 3A). To evaluate the effect of EGT we also included a group of mice, vaccinated i.m. without EGT with the construct RBD-S2′ (Figure 3A, group 2). Mice were routinely checked for health status during the experimental period: no adverse effects nor differences in body weight among groups were observed.

As a quality control of the EGT procedure, we have estimated the current flow in all the vaccinated mice, reporting global computed currents after the first (prime) and the second (boost) EGT. As expected, the assessed current values were homogeneous and dependent solely on the leg radius of each mouse (Figure 3B). No statistically significant differences were found.

### 3.4 Antibody response to DNA vaccination

**Figure 4:**
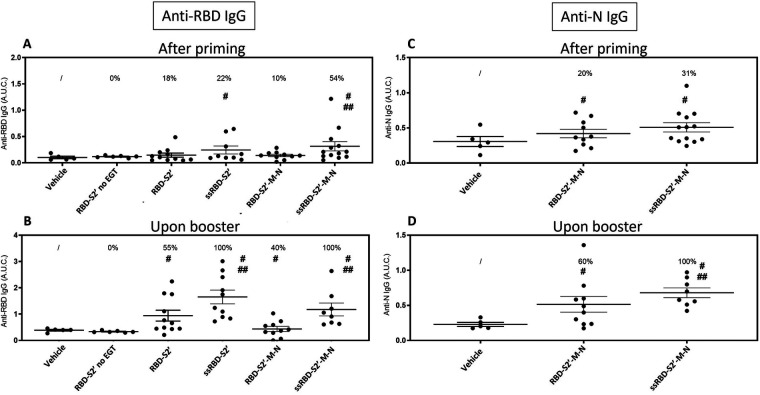
The secretory sequence from PGIP increased antibody response to DNA vaccination. Sera from mice immunized with the indicated construct followed by EGT were collected 2 weeks after priming **(A, C)** or after booster **(B, D)** and analyzed by ELISA to assess the presence of anti-RBD **(A, B)** or anti-N **(C, D)** IgG. Data for mice vaccinated with the RBD-S2′ construct in the absence of EGT (RBD-S2′ no EGT) were also included. The Area Under the Curve (A.U.C.) of O.D. values vs. serum serial dilutions plots were calculated with the GraphPad Prism software for each individual mouse (single dots). For each group the mean A.U.C. (horizontal bar) ± standard error is also shown (mice/group: 5 for Vehicle, 6 for RBD-S2′ no EGT, 11 for RBD-S2′, 9 for ss-RBD-S2′, 10 for RBD-S2′-M-N, 13 for ss-RBD-S2′-M-N). The numbers above each group represent the percentage of positive mice, defined as the percentage of mice whose A.U.C. value exceeded the mean A.U.C. value of the vehicle group (non-immunized mice) by more than 2 SD. #, *p* < 0.05 when compared with the vehicle group; ##, *p* < 0.05 when compared with corresponding construct devoid of PGIPss.

Two weeks after priming (day 14) and after boost (day 35), sera were collected from immunized mice and analyzed by ELISA to assess anti-RBD and anti-N IgG responses. All DNA constructs revealed to be immunogenic, as mice generated an antigen-specific antibody response (Figure 4). Results showed that, in the absence of EGT, the injection with the RBD-S2′ construct did not induce an appreciable response (Figure 4A), even after the second immunization (boost) (Figure 4B), compared to the vehicle group. At variance, as expected, when mice injected with RBD-S2′ DNA underwent EGT, they produced a higher RBD-specific IgG response (upon booster mean AUC ± S.E.: 0.9410 ± 0.2069 vs. 0.3358 ± 0.0145), with 50% of mice showing seroconversion upon booster. Addition of the PGIPss to the RBD-S2′ construct (ssRBD-S2′ vs. RBD-S2′) further increased the anti-RBD IgG response (upon booster mean AUC ± S.E.: 1.6515 ± 0.2603 vs. 0.9410 ± 0.2069), with 100% of mice being positive upon booster. Of note the PGIPss also increased the antibody (Ab) response to RBD when mice were injected with the RBD-S2′-M-N construct (upon booster mean AUC ± S.E. was 1.1759 ± 0.2448 for ssRBD-S2′-M-N vs. 0.4361 ± 0.0969 for RBD-S2′-M-N) with 100% of positive mice upon booster.

Sera from mice groups injected with the constructs containing the RBD-S2′-M-N sequence were also analyzed for the presence of anti-N IgG (Figures 4C,D). Results showed that when mice were immunized with the RBD-S2′-M-N construct, the addition of the secretory sequence improved the Ab response (upon booster mean AUC ± S.E. 0.6789 ± 0.0691 for ssRBD-S2′-M-N vs. 0.5146 ± 0.1120 for RBD-S2′-M-N) increasing the frequency of positive mice from 60%–100%.

Altogether, these results showed that EGT is required to induce an appreciable Ab response to RBD and that boosting the animals with a second injection of the constructs followed by EGT increases IgG response (AUC values). More strikingly, only when the PGIPss is included in the DNA constructs, all the mice (100%) produced an IgG response towards both RBD and N proteins.

### 3.5 CD4T cell response to DNA vaccination

**Figure 5:**
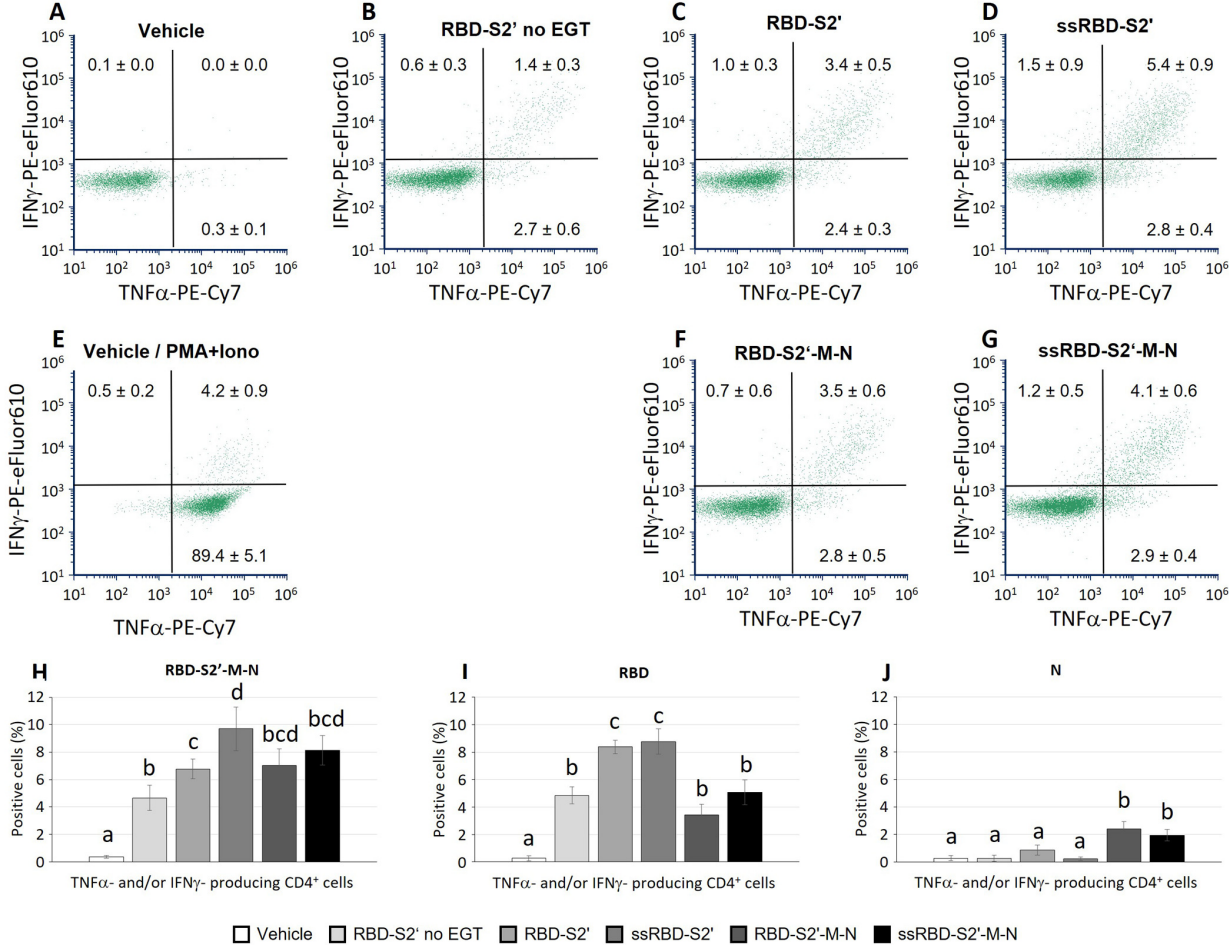
The PGIP secretory sequence increased the induction of polyfunctional CD4 cells in response to vaccination. Spleen cells from mice immunized with the indicated construct followed by EGT were stimulated *in vitro* with either the RBD-S2′-M-N **(A-H)**, or RBD **(I)** or N **(J)** protein and analyzed by flow cytometry. Stimulation of cells from the vehicle group (mice immunized with water, A) with PMA and ionomycin is also shown **(E)** Data from mice vaccinated with the RBD-S2′ construct in the absence of EGT (RBD-S2′ no EGT) were also included **(B)**. Images in panels A-G show a representative dot plot for each group. At variance, numbers in dot plots quadrants represent the average percentage of 6-10 mice/group (means ± S.E.; mice/group: 5 for Vehicle, 6 for RBD-S2′ no EGT, 9 for RBD-S2′, 9 for ss-RBD-S2′, 10 for RBD-S2′-M-N, 10 for ssRBD-S2′-M-N) for CD4 cells producing IFN-ɣ (upper left quadrants), TNF-α (lower right) or both IFN-ɣ and TNF-α (upper right; poly-functional CD4 cells). Columns represent percentage of poly-functional CD4 cells in cultures stimulated with RBD-S2′-M-N **(H)**, RBD **(I)** or N **(J)** protein. Values are means of 6-10 mice/group [as in **(A-G)**] ± S.E. Different letters above the bars indicate statistically significant differences between groups (*p* < 0.05, ANOVA followed by Tukey-Kramer test). Groups that share the same letter are not significantly different.

Two weeks after boost, mice were sacrificed, and spleen cells collected to evaluate cell-mediated responses. After *in vitro* stimulation with the chimeric RBD-S2′-M-N protein, spleen cells were analyzed by flow cytometry using the hierarchical gating strategy shown in Supplementary Figure S5. Mice immunization induced the activation of CD4 cells producing only TNF-α (in all groups), only IFN-ɣ (fewer cells) or both IFN-ɣ and TNF-α (poly-functional CD4 cells). Results show that mice injected with the RBD-S2′ construct in the absence of EGT (RBD-S2′ no EGT in Figure 5B) generated a response with 2.7% of CD4 cells expressing only TNF-α and 1.4% both TNF-α and IFN-ɣ. CD4 cells expressing only IFN-ɣ were barely detectable (0.6%). In mice vaccinated with RBD-S2′ construct followed by EGT (Figure 5C), the specific CD4T cell response increased, especially for cells expressing both TNF-α and IFN-ɣ compared with DNA vaccination in the absence of EGT (3.4% vs. 1.4%, Figure 5C vs. 5B). In electroporated animals, vaccination with either RBD-S2′ (Figure 5C) or RBD-S2′-M-N (Figure 5F) gave rise to comparable responses, suggesting that the CD4T cell response was primarily toward the RBD epitope. The addition of the PGIPss further increased the CD4T cell response (Figure 5D,G). In particular, CD4 cells expressing both TNF-α and IFN-ɣ raised from 3.4% for the RBD-S2′ (Figure 5C) to 5.4% for ssRBD-S2′ (Figure 5D) and from 3.5% for RBD-S2′-M-N (Figure 5F) to 4.1% for ssRBD-S2′-M-N (Figure 5G). The percentage of CD4 cells expressing only IFN-ɣ also increased (1.0% for RBD-S2′, Figure 5C, vs. 1.5% for ssRBD-S2′, Figure 5D, and 0.7% for RBD-S2′-M-N, Figure 5F, vs. 1.2% for ssRBD-S2′-M-N, Figure 5G). CD4 cells from vehicle-injected mice (negative control) showed absent/negligible expression of IFN-ɣ and TNF-α (Figure 5A) in response to the RBD-S2′-M-N protein, whereas they produced cytokines when stimulated with PMA + ionomycin (positive control, Figure 5E).

In parallel cultures, spleen cells from all mice were stimulated with either the RBD or the N protein to assess the response to the main peptides included in the chimeric vaccine constructs. Results in Figures 5H–J show the percentage of antigen-specific CD4 cells, that is determined by the CD4 cells producing at least one of the two analyzed cytokines (IFN-ɣ and/or TNF-α) in response to RBD-S2′-M-N (Figure 5H), RBD (Figure 5I) or N (Figure 5J) protein. Results show that the CD4 cell response is mainly towards the RBD component of the constructs, since we recorded a higher response when spleen cells are stimulated with the RBD peptide (Figure 5I) and a lower response when stimulated with the N protein (Figure 5J). The effects of the presence of PGIPss in enhancing CD4 cell responses were evident also when cells were stimulated with RBD alone (Figure 5I), and not only with the full-length RBD-S2-M-N protein (Figure 5H).

### 3.6 CD8T cell response to DNA vaccination

**Figure 6:**
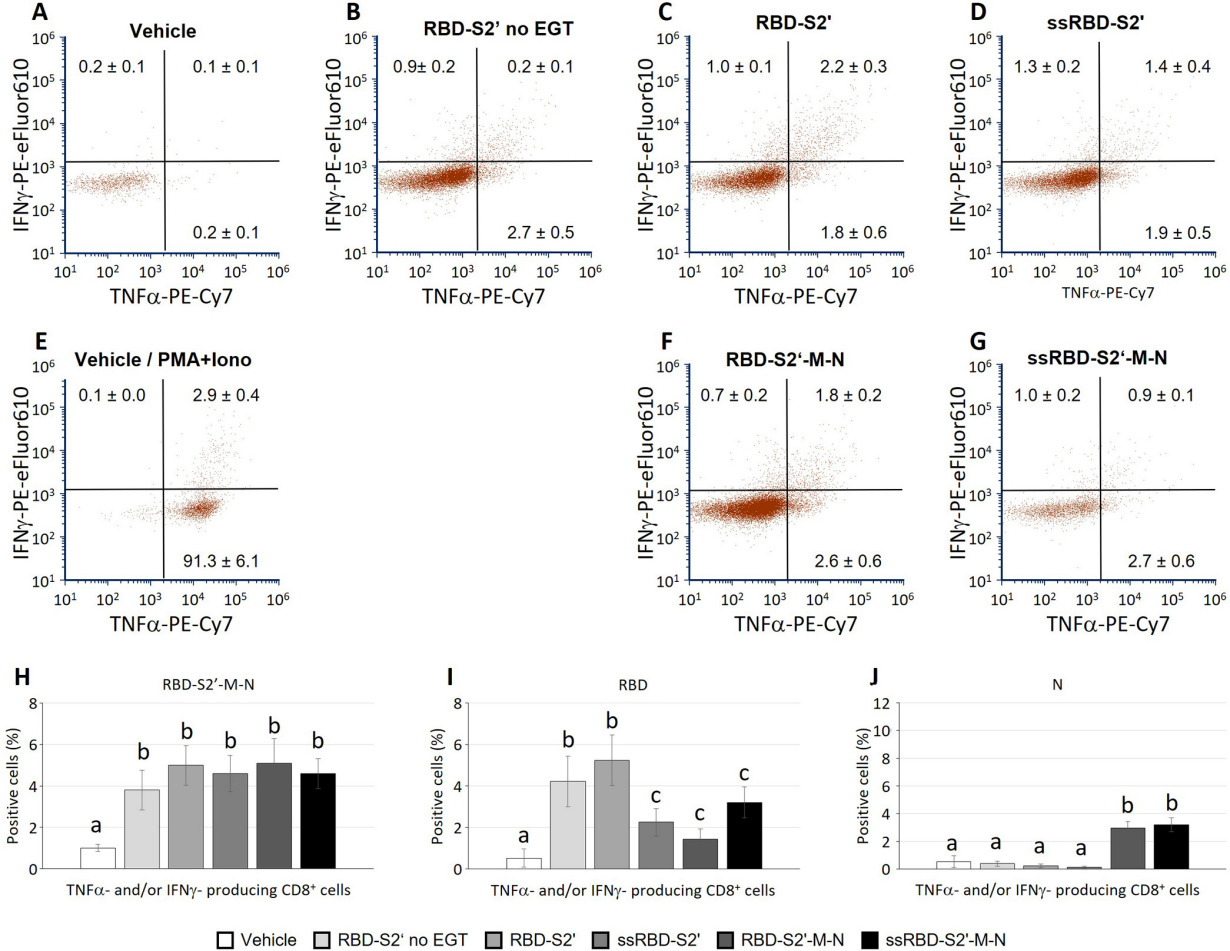
The PGIP secretory sequence reduced the induction of polyfunctional CD8 cells towards RBD in response to vaccination, not single cytokine-producing CD8 cells. Spleen cells from mice immunized with the indicated construct followed by EGT were stimulated as described in Figure 5. Images in panels **(A-G)** show a representative dot plot for each group. At variance, numbers in dot plots quadrants represent the average percentage of 5-9 mice/group (means ± S.E.; mice/group: 5 for Vehicle, 6 for RBD-S2′ no EGT, 9 for RBD-S2′, 9 for ss-RBD-S2′, 9 for RBD-S2′-M-N, 9 for ssRBD-S2′-M-N) for CD8 cells producing IFN-ɣ (upper left quadrants within each dot plot), TNF-α (lower right quadrant) or both IFN-ɣ and TNF-α (upper right quadrant; poly-functional CD8 cells). Columns represent percentage of poly-functional CD8 cells in cultures stimulated with RBD-S2′-M-N **(H)**, RBD **(I)** or N **(J)** protein. Values are means of 6-10 mice/group [as in **(A-G)**] ± S.E. Different letters above the bars indicate statistically significant differences between groups (*p* < 0.05, ANOVA followed by Tukey-Kramer test). Groups that share the same letter are not significantly different.

CD8 cells contained in the spleen cell suspension, stimulated and analyzed as described above, produced TNF-α and/or IFN-ɣ. Results in Figure 6B show that mice injected with the RBD-S2′ construct in absence of EGT (RBD-S2′ no EGT in Figure 6B) generated a response with 2.7% or 0.9% of CD8 cells expressing only TNF-α or IFN-ɣ, respectively. CD8 cells expressing both cytokines were barely detectable (0.2%). In mice vaccinated with RBD-S2′ construct followed by EGT, the CD8T cell response increased, especially for cells expressing both TNF-α and IFN-ɣ compared with DNA vaccination in the absence of EGT (2.2% vs. 0.2%, Figure 6C vs. Figure 6B). In electroporated mice, vaccination with either the RBD-S2′ or the RBD-S2′-M-N construct gave rise to similar responses, suggesting that the CD8T cell response was also primarily toward the RBD epitope (Figures 6C,F). Unexpectedly, the addition of the PGIPss reduced the percentage of CD8T cells expressing both TNF-α and IFN-ɣ (Figures 6D,G). In particular, CD8 cells expressing both TNF-α and IFN-ɣ dropped from 2.2% for RBD-S2′ (Figure 6C) to 1.4% for ssRBD-S2′ (Figure 6D), and from 1.8% for RBD-S2′-M-N (Figure 6F) to 0.9% for ss-RBD-S2′-M-N (Figure 6G). The percentage of CD8 cells expressing only one of the two cytokines (IFN-ɣ or TNF-α alone) was not affected. CD8 cells from vehicle-injected mice (negative control group) showed absent/negligible expression of IFN-ɣ and TNF-α in response to the RBD-S2′-M-N protein (Figure 6A), whereas they produced cytokines when stimulated with PMA + ionomycin (positive control, Figure 6E).

In parallel cultures, spleen cells were stimulated with either the RBD or the N protein to assess the response to the specific main peptides included in the chimeric vaccine constructs. Results in Figure 6H-J represent the percentage of CD8 cells that produced at least one of the two analyzed cytokines (IFN-ɣ and/or TNF-α) in response to RBD-S2′-M-N (Figure 6H), RBD (Figure 6I) or N protein (Figure 6J). As observed for the CD4 response, the CD8 cell response is also mainly towards the RBD component (Figure 6I), the response being higher when spleen cells are stimulated with the RBD peptide (Figure 6I) and lower, although appreciable, when stimulated with the N protein (Figure 6J).

## 4 Discussion

DNA vaccines deliver sequences encoding antigens/epitopes into host cells, enabling their production *in vivo* with many associated advantages: stimulation of both cellular and humoral immune responses; possibility to tailor and direct immunity against either a specific target protein or different epitopes (multi-epitopes vaccines); modulation of the specificity and type of the immune response; rapidity and ease of production and storage (1, 35–38).

Different strategies have been developed for the design of anti-SARS-CoV-2 broad range DNA vaccines (1, 39–42). The DNA platform offers the opportunity to combine several epitopes of different viral proteins, avoiding the use of large proteins that can contain unnecessary epitopes possibly leading to increased chances of undesirable responses (39). However, the correct folding of the chimeric protein is crucial, and the main drawback of this technology is the scarce immunogenicity of the resulting constructs (11, 43–45).

In this study, we report the rational design, expression, and immunogenicity assessment of four SARS-CoV-2 DNA vaccine constructs encoding chimeric proteins that incorporate the receptor-binding domain (RBD) of the spike (S) protein and selected immunogenic epitopes from the S, membrane (M) and nucleocapsid (N) proteins. Our results demonstrate that the inclusion of a plant-derived secretory signal peptide (PGIPss) significantly enhances antigen expression and immunogenicity of vaccines delivered by electro-gene transfer (EGT).

Secretory and membrane protein sorting critically depends on signal sequences. From bacteria to eukaryotes, protein sorting is mostly conserved, despite possible differences in signal sequence length and amino acid content. Our previous findings showed that the plant signal sequence PGIPss (ss) can trigger secretion in mammalian cells as well, increasing the immunogenicity of fused antigens that would otherwise be “immunologically weak” (18, 46). To enhance the immune response to the developed SARS-CoV-2 DNA-based vaccines, we used EGT, a clinical stage of research technology improving DNA delivery and causing local inflammation (1, 4, 6–8).

The constructs we have created share the receptor binding domain (RBD) of the S protein of SARS-CoV-2 virus, responsible of the viral binding to the ACE2 receptor, together with an additional epitope of the S protein that includes the S2′ cleavage site (furin-like), responsible for the viral fusion with cellular membranes (RBD-S2′) (26, 27). The RBD from the B.1.351 variant was chosen to induce antibodies against a highly immune-evasive variant (24). The inclusion of the S2′ peptide from internal fusion peptide regions further expands the immunological relevance of the construct, potentially targeting early stages of viral entry (26, 27).

We verified that the RBD-S2′ protein was successfully expressed in mammalian cells (Figure 2A, panel b), and it was able to stimulate a robust antibody response when administered in mice by i.m. injection followed by EGT. When the construct was delivered in absence of EGT, the response was weaker, confirming that the EGT is an effective “adjuvant” for DNA vaccines (47). When the PGIPss was fused upstream the constructs, the expression of the recombinant protein in mammalian cells was higher (Figure 2A, panel c) and the anti-RBD antibody response increased in vaccinated mice, confirming and extending our previous findings on the use of this sequence to potentiate antigen-specific immune responses (18). Noteworthy, the antigen-specific CD4T cell response was also enhanced by the PGIPss.

Alongside the assessment of responses to the RBD-S2′ and ssRBD-S2′ chimeric proteins, we investigated the possibility to trigger a specific immune response against other viral epitopes that are more conserved among coronaviruses and able to induce protection by cross-reactive CD4^+^ and CD8^+^ T cells against related viral recurrence (19, 22, 23). We included multi-epitope sequences from the M and N proteins (obtaining the constructs named RBD-S2′-M-N and ssRBD-S2′-M-N). These structural proteins, particularly the N protein, have been associated with strong T cell responses in convalescent individuals and are highly conserved among SARS-CoV-2 variants (31, 48), making them attractive candidates for inclusion in next-generation vaccines.

Both M and N proteins are well studied structural proteins that present both CD4 and CD8 epitopes (28–31). For the M protein we selected a short CTL peptide (9 aa: ATSRTLSYY, Figure 1 and Supplementary Figure S2A) that was previously described as the unique probable non allergen epitope in the M protein (28). For N protein, we selected a long peptide (75 aa) that includes 3 immunogenic epitopes previously described (30, 31). The first evidence in terms of expression in mammalian cells indicated that the RBD-S2′-M-N protein was successfully expressed (Figure 2A, panel d), and this occurred at similar levels of RBD-S2′(Figure 2A, panel b). Also in this case, the addition of the PGIPss led to a significant increase of expression that was reflected in a significant boost on immunogenicity (Figure 2A, panel e). However, the antibody stimulation was weaker compared to its counterpart without the M and N epitopes (construct ssRBD-S2′) (Figure 4). This can be due to a not proper folding of the RBD-S2′-M-N protein or to a weaker expression of the construct *in vivo* (which we have not verified *in vivo*). To evaluate the integrity of the chimeric RBD-S2′M-N protein and the eventual presence of specific responses against the long N epitope, we performed an ELISA using the full-length N protein as coating antigen. We observed an antibody response against the N epitope that, again, is higher in the presence of the PGIPss. In this case we also observed a higher background response, probably due to unspecific cross-reactions of mice sera against the SARS-CoV-2 N protein or to putative contaminants present in the N protein commercial preparation. We also performed immunofluorescence microscopy on transfected mammalian cells using an anti-N polyclonal antibody instead of the anti-RBD one and we observed an increase of expression of the chimeric protein in the presence of the PGIPss (Figure 2B panel h vs. panel g). The detection of the RBD-S2′-M-N and ssRBD-S2′-M-N with the anti-N antibody (Figure 2B) was lower compared to that obtained with the anti-RBD-antibody (Figure 2A). We cannot state if this result is due either to a lower affinity of the polyclonal anti-N protein antibody (obtained against the full-length N protein) toward the RBD-S2-M-N-containing constructs (including only 75 aa of N) compared to that of the anti-RBD antibody or to an incorrect folding of the C-terminal part of the recombinant multi-epitope proteins. This limitation could be addressed in future experiments by including a longer peptide or the full-length N (or M protein) to improve its “visibility” to the immune system. An alternative strategy could include the co-vaccination with different constructs, each harboring a single gene/Long epitope.

When cells were stimulated *in vitro* with the whole RBD-S2′-M-N protein, spleen cells from mice immunized with EGT and either RBD-S2′ or RBD-S2′-M-N gave rise to comparable CD4 and CD8T cell responses (Figure 5, 6). Cells were also stimulated with either the RBD-S2′ or N protein for comparison with the whole RBD-S2′-M-N protein (specific response to M epitope alone was not assessed). The results showed that CD4 and CD8T cell responses were mainly towards the RBD component of the constructs, suggesting that, in our settings, epitopes other than those from RBD (including M) did not contribute in a relevant way.

Antibody response toward protein antigens is sustained by CD4 helper T cells which induce B cells to undergo differentiation, isotype switch and affinity maturation in germinal centers, resulting in the production of high affinity antigen-specific IgG and generation of long-lived plasma cells. Mice immunization with our constructs induced an antigen-specific CD4T cell response as revealed by the ability of these cells to express TNF-α and/or IFN-ɣ when stimulated *in vitro* with the antigen. Noteworthy, EGT doubled the frequency of antigen-specific CD4 cells, in particular of cells expressing both TNF-α and IFN-ɣ compared with DNA vaccination alone. A further increase in TNF-α- and IFN-ɣ- producing CD4 cells was observed when mice were immunized by constructs containing the PGIPss.

In a previous work we demonstrated that fusion of viral antigens from the human papilloma virus (HPV) to the PGIPss triggers effective immune responses in mice (18). Several studies showed that signal sequences have important effects on protein expression, maturation, compartmentalization and release, affecting epitope immunogenicity and quality of immune responses, with effects also depending on the route of administration (49, 50). Although of plant origin, PGIPss drives the encoded protein to the endoplasmic reticulum and Golgi apparatus in mammalian cells, a finding that could explain why in the current study PGIPss increased both antibody and CD4 cell responses. Indeed, secreted antigens can be up taken by the (few) dendritic cells (DCs) localized in the muscle which, upon maturation induced by local inflammation, migrate to the paracortex of draining lymph nodes. Secreted antigens can also reach lymph nodes directly through lymphatics. In lymph nodes, soluble antigens can be taken up by DCs and macrophages and/or be recognized by the membrane-associated antibody of B cells. In lymph node paracortex, antigen-loaded DCs prime naïve CD4T cells generating effector helper T cells which in turn sustain B cell activation and differentiation to antibody-producing cells (51–53).

Although protection from viral infection is ensured by neutralizing high affinity antibodies, cytotoxic CD8T cells largely contribute to the protection from severe infection and reinfection. Indeed, an early development of CD8T cell responses to SARS-CoV-2 correlates with mild disease and pathogen clearance (54). Our constructs were able to elicit a polyfunctional CD8T cell response as revealed by the expression of multiple cytokines (TNF-α and IFN-ɣ). Mice were injected i.m., a route previously shown to induce stronger T cell responses compared with intradermal or subcutaneous routes (55, 56). The i.m. route was also reported to be a better inducer of polyfunctional CD4T-cell, in particular of Th1 IFN-ɣ-secreting CD4 cells which could sustain CD8T cell responses (55, 57). These features make the i.m route suitable to induce cell-mediated immune responses.

It is known that DNA vaccination via i.m. injection, especially when accompanied by EGT, can result in myocytes transfection. However, EGT not only increases the chances of effective transfection and protein expression, but it can also induce apoptosis in muscles cells. Although the muscular tissue is relatively poor of dendritic cells, EGT-induced tissue damage raises an inflammatory response with consequent immune cell recruitment and phagocytosis of apoptotic cells by dendritic cells, leading to cross-presentation (8). Cross-presentation is central in inducing an antigen-specific immune response towards viruses (58).

The aim of vaccination is to prevent infections in vaccinated subjects by inducing neutralizing antibodies. However, potentiation of antibody responses vs. cell-mediated responses requires the exploitation of different pathways and the right equilibrium may depend on route of administration, antigen type/epitope specificities, adjuvant, and other factors. PGIPss, as discussed above, increased the antibody response as well as the CD4T cell response in i.m. immunized mice therefore potentiating the humoral response. At variance, the addition of PGIPss reduced the induction of CD8 cells secreting both TNF-α and IFN-ɣ (polyfunctional CD8 cells), although without affecting the percentage of CD8 cells expressing only one of the two cytokines (IFN-ɣ or TNF-α alone). Many details in CD8T cell priming by DCs, especially via cross-presentation with its complex and multiple ways, remain unsolved and mechanistic data were mainly obtained *in vitro* (58). It is therefore difficult to understand the mechanisms affected by the PGIPss in this context. By promoting the secretory route, PGIPss might reduce the availability of the antigen for other pathways (cytosol) and/or promote peptide degradation (delayed degradation favor cross-presentation in DCs). Moreover, the reduction in polyfunctional CD8 cells was observed mainly in response to the stimulation with RBD alone (not with the whole RBD-S2′-M-N protein; Figure 6H vs. Figure 6I). In future studies the role of PGIPss in the response towards different epitopes (including the M CD8 epitope we did not analyze in the current study) will help to understand the relevance of this effect.

## 5 Limitations

While promising, our study has limitations. First, although we found that the PGIPss potentiates the antibody response we did not assess if the induced antibodies had functional neutralizing activity. Second, the expression of the different chimeric proteins was assessed in HEK-293 cells only, and not in vaccinated mice, making it more difficult the understanding of the fine tuning of immune responses. Additionally, the responses were evaluated against the RBD-S′-M-N full length protein, or against the RBD domain or against the full-length N proteins. The responses against the M and the S2′ epitopes are missing.

## 6 Conclusions

Collectively, our results support the feasibility of a DNA-based multi-epitope vaccine that combines the benefits of variant-specific spike antigens with conserved epitopes from internal structural proteins. The construct design and the inclusion of a plant-derived signal peptide provide a novel approach to enhance expression and immunogenicity.

Our data reinforce the need to optimize antigen presentation, delivery methods, and epitope selection to fully harness the potential of DNA vaccine platforms. Further investigations into optimizing the secretory signal and balancing antigen trafficking between MHC-I and MHC-II pathways could refine the vaccine's ability to induce balanced humoral and cellular immunity against different viral epitopes.

## Data Availability

The original contributions presented in the study are included in the article/Supplementary Material, further inquiries can be directed to the corresponding authors.
