# Supplementary material for: A plant secretory sequence enhances immunogenicity of electroporated COVID-19 DNA vaccines
Source: Front Med Technol. 2025 Jul 14;7:1597179. doi: 10.3389/fmedt.2025.1597179 (PMC12301313; doi:10.3389/fmedt.2025.1597179)
Supplement: Supplementary file 1 [file Datasheet1.pdf]

Supplementary Material

S protein

```
MFVFLVLLPLVSSQCVNLTTRTQLPPAYTNSFTRGVYYPDKVFRSSVLHSTQDLFLPFFS   60
NVTWFHAIHVSGTNGTKRFDNPVLPFNDGVYFASTEKSNIIRGWIFGTTLDSKTQSLLIV  120
NNATNVVIKVCEFQFCNDPFLGVYYHKNNKSWMESEFRVYSSANNCTFEYVSQPFLMDLE  180
GKQGNFKNLREFVFKNIDGYFKIYSKHTPINLVRDLPQGFSALEPLVDLPIGINITRFQT  240
LLALHRSYLTPGDSSSGWTAGAAAYYVGYLQPRTFLLKYNENGTITDAVDCALDPLSETK  300
CTLKSFTVEKGIYQTSNFRVQPTESIVRFPNITNLCPFGEVFNATRFASVYAWNRKRISN  360
CVADYSVLYNSASFSTFKCYGVSPTKLNDLCFTNVYADSFVIRGDEVRQIAPGQTGKIAD  420
YNYKLPDDFTGCVIAWNSNNLDSKVGGNYNYLYRLFRKSNLKPFERDISTEIYQAGSTPC  480    RBD
NGVEGFNCYFPLQSYGFQPTNGVGYQPYRVVVLSFELLHAPATVCGPKKSTNLVKNKCVN  540
FNFNGLTGTGVLTESNKKFLPFQQFGRDIADTTDAVRDPQTLEILDITPCSFGGVSVITP  600
GTNTSNQVAVLYQDVNCTEVPVAIHADQLTPTWRVYSTGSNVFQTRAGCLIGAEHVNNSY  660
ECDIPIGAGICASYQTQTNSPRRARSVASQSIIAYTMSLGAENSVAYSNNSIAIPTNFTI  720
SVTTEILPVSMTKTSVDCTMYICGDSTECSNLLLQYGSFCTQLNRALTGIAVEQDKNTQE  780
VFAQVKQIYKTPPIKDFGGFNFSQILPDPSKPSKRSFIEDLLFNKVTLADAGFIKQYGDC  840    S2'
LGDIAARDLICAQKFNGLTVLPPLLTDEMIAQYTSALLAGTITSGWTFGAGAALQIPFAM  900
QMAYRFNGIGVTQNVLYENQKLIANQFNSAIGKIQDSLSSTASALGKLQDVVNQNAQALN  960
TLVKQLSSNFGAISSVLNDILSRLDKVEAEVQIDRLITGRLQSLQTYVTQQLIRAAEIRA  1020
SANLAATKMSECVLGQSKRVDFCGKGYHLMSFPQSAPHGVVFLHVTYVPAQEKNFTTAPA  1080
ICHDGKAHFPREGVFVSNGTHWFVTQRNFYEPQIITTDNTFVSGNCDVVIGIVNNTVYDP  1140
LQPELDSFKEELDKYFKNHTSPDVDLGDISGINASVVNIQKEIDRLNEVAKNLNESLIDL  1200
QELGKYEQYIKWPWYIWLGFIAGLIAIVMVTIMLCCMTSCCSCLKGCCSCGSCCKFDEDD  1260
SEPVLKGVKLHYT  1273
```

Figure S1. COVID-19 spike protein epitopes RBD and S2' included in the genetic constructs.

The RBD (aa 319-541) is highlighted in yellow and harbours the 3 mutations (K417>N; E484 >K; N501>Y) of the B.1.351 variant strain (24).

The S2' epitope is highlighted in grey and includes the S2' cleavage site (furin-like) (KRSF, in blue, aa 815-818) and upstream and downstream amino acids, comprising amino acids of the internal fusion peptide (IFP, underlined) (26) (aa 817-834).

1) RBD-S2’

ATCATGCGCGTGCAGCCTACCGAGTCCATCGTGCGCTTCCCTAACATCACCAACCTGTGCCCTTTCGGTGAGGTGTTCAACGCTACCCGCTTCGCTTCCGTGTACGCTTGGAACCGCAAGCGCATCTCCA
ACTGCGTGGCTGACTACTCCGTGCTGTACAACTCCGCTTCCTTCTCCACCTTCAAGTGCTACGGTGTGTCCCCTACCAAGCTGAACGACCTGTGCTTCACCAACGTGTACGCTGACTCCTTCGTGATCCGC
GGTGACGAGGTGCGCCAGATCGCTCCTGGTCAGACCGGTAACATCGCTGACTACAACTACAAGCTGCCTGACGACTTCACCGGTTGCGTGATCGCTTGGAACTCCAACAACCTGGACTCCAAGGTGGGT
GGTAACTACAACTACCTGTACCGCCTGTTCCGCAAGTCCAACCTGAAGCCTTTCGAGCGCGACATCTCCACCGAGATCTACCAGGCTGGTTCCACCCCTTGCAACGGTGTGAAGGGTTTCAACTGCTACT
TCCCTCTGCAGTCCTACGGTTTCCAGCCTACCTACGGTGTGGGTTACCAGCCTTACCGCGTGGTGGTGCTGTCCTTCGAGCTGCTGCACGCTCCTGCTACCGTGTGCGGTCCTAAGAAGTCCACCAACCTG
GTGAAGAACAAGTGCGTGAACTTCGCTGCTTACCCTTCCAAGCCTTCCAAGCGCTCCTTCATCGAGGACCTGCTGTAA

2) ssRBD-S2’

ATCATGACCCAGTTCAACATCCCTGTGACCATGTCCTCCTCCCTGTCCATCATCCTGGTGATCCTGGTGTCCCTGCGCACCGCTCTGTCCATGCGCGTGCAGCCTACCGAGTCCATCGTGCGCTTCCCTAA
CATCACCAACCTGTGCCCTTTCGGTGAGGTGTTCAACGCTACCCGCTTCGCTTCCGTGTACGCTTGGAACCGCAAGCGCATCTCCAACTGCGTGGCTGACTACTCCGTGCTGTACAACTCCGCTTCCTTCT
CCACCTTCAAGTGCTACGGTGTGTCCCCTACCAAGCTGAACGACCTGTGCTTCACCAACGTGTACGCTGACTCCTTCGTGATCCGCGGTGACGAGGTGCGCCAGATCGCTCCTGGTCAGACCGGTAACAT
CGCTGACTACAACTACAAGCTGCCTGACGACTTCACCGGTTGCGTGATCGCTTGGAACTCCAACAACCTGGACTCCAAGGTGGGTGGTAACTACAACTACCTGTACCGCCTGTTCCGCAAGTCCAACCT
GAAGCCTTTCGAGCGCGACATCTCCACCGAGATCTACCAGGCTGGTTCCACCCCTTGCAACGGTGTGAAGGGTTTCAACTGCTACTTCCCTCTGCAGTCCTACGGTTTCCAGCCTACCTACGGTGTGGGT
TACCAGCCTTACCGCGTGGTGGTGCTGTCCTTCGAGCTGCTGCACGCTCCTGCTACCGTGTGCGGTCCTAAGAAGTCCACCAACCTGGTGAAGAACAAGTGCGTGAACTTCGCTGCTTACCCTTCCAAGC
CTTCCAAGCGCTCCTTCATCGAGGACCTGCTGTAA

3) RBD-S2’-M-N

ATCATGCGCGTGCAGCCTACCGAGTCCATCGTGCGCTTCCCTAACATCACCAACCTGTGCCCTTTCGGTGAGGTGTTCAACGCTACCCGCTTCGCTTCCGTGTACGCTTGGAACCGCAAGCGCATCTCCA
ACTGCGTGGCTGACTACTCCGTGCTGTACAACTCCGCTTCCTTCTCCACCTTCAAGTGCTACGGTGTGTCCCCTACCAAGCTGAACGACCTGTGCTTCACCAACGTGTACGCTGACTCCTTCGTGATCCGC
GGTGACGAGGTGCGCCAGATCGCTCCTGGTCAGACCGGTAACATCGCTGACTACAACTACAAGCTGCCTGACGACTTCACCGGTTGCGTGATCGCTTGGAACTCCAACAACCTGGACTCCAAGGTGGGT
GGTAACTACAACTACCTGTACCGCCTGTTCCGCAAGTCCAACCTGAAGCCTTTCGAGCGCGACATCTCCACCGAGATCTACCAGGCTGGTTCCACCCCTTGCAACGGTGTGAAGGGTTTCAACTGCTACT
TCCCTCTGCAGTCCTACGGTTTCCAGCCTACCTACGGTGTGGGTTACCAGCCTTACCGCGTGGTGGTGCTGTCCTTCGAGCTGCTGCACGCTCCTGCTACCGTGTGCGGTCCTAAGAAGTCCACCAACCTG
GTGAAGAACAAGTGCGTGAACTTCGCTGCTTACCCTTCCAAGCCTTCCAAGCGCTCCTTCATCGAGGACCTGCTGGCTGCTTACGCTACCTCCCGCACCCTGTCCTACTACGCTGCTTACAAGGCTTACA
ACGTGACCCAGGCTTTCGGTCGCCGCGGTCCTGAGCAGACCCAGGGTAACTTCGGTGACCAGGAGCTGATCCGCCAGGGTACCGACTACAAGCACTGGCCTCAGATCGCTCAGTTCGCTCCTTCCGCTT
CCGCTTTCTTCGGTATGTCCCGCATCGGTATGGAGGTGACCCCTTCCGGTACCTGGCTGACCTACACCGGTGCTATCAAGCTGGACTAA

4) ssRBD-S2’-M-N

ATCATGACCCAGTTCAACATCCCTGTGACCATGTCCTCCTCCCTGTCCATCATCCTGGTGATCCTGGTGTCCCTGCGCACCGCTCTGTCCATGCGCGTGCAGCCTACCGAGTCCATCGTGCGCTTCCCTAA
CATCACCAACCTGTGCCCTTTCGGTGAGGTGTTCAACGCTACCCGCTTCGCTTCCGTGTACGCTTGGAACCGCAAGCGCATCTCCAACTGCGTGGCTGACTACTCCGTGCTGTACAACTCCGCTTCCTTCT
CCACCTTCAAGTGCTACGGTGTGTCCCCTACCAAGCTGAACGACCTGTGCTTCACCAACGTGTACGCTGACTCCTTCGTGATCCGCGGTGACGAGGTGCGCCAGATCGCTCCTGGTCAGACCGGTAACAT
CGCTGACTACAACTACAAGCTGCCTGACGACTTCACCGGTTGCGTGATCGCTTGGAACTCCAACAACCTGGACTCCAAGGTGGGTGGTAACTACAACTACCTGTACCGCCTGTTCCGCAAGTCCAACCT
GAAGCCTTTCGAGCGCGACATCTCCACCGAGATCTACCAGGCTGGTTCCACCCCTTGCAACGGTGTGAAGGGTTTCAACTGCTACTTCCCTCTGCAGTCCTACGGTTTCCAGCCTACCTACGGTGTGGGT
TACCAGCCTTACCGCGTGGTGGTGCTGTCCTTCGAGCTGCTGCACGCTCCTGCTACCGTGTGCGGTCCTAAGAAGTCCACCAACCTGGTGAAGAACAAGTGCGTGAACTTCGCTGCTTACCCTTCCAAGC
CTTCCAAGCGCTCCTTCATCGAGGACCTGCTGGCTGCTTACGCTACCTCCCGCACCCTGTCCTACTACGCTGCTTACAAGGCTTACAACGTGACCCAGGCTTTCGGTCGCCGCGGTCCTGAGCAGACCCA
GGGTAACTTCGGTGACCAGGAGCTGATCCGCCAGGGTACCGACTACAAGCACTGGCCTCAGATCGCTCAGTTCGCTCCTTCCGCTTCCGCTTTCTTCGGTATGTCCCGCATCGGTATGGAGGTGACCCCT
TCCGGTACCTGGCTGACCTACACCGGTGCTATCAAGCTGGACTAA

Figure S2. Nucleotide sequences of the genetic constructs shown in Fig. 1. Kozak sequence (ATC) upstream the first ATG is in bold.

A) **M protein**

MADSNGTITVEELKKLLEQWNLVIGFLFLTWICLLQFAYANRNRFLYIIKLIFLWLLWPVTLACFVLAAV
YRINWITGGIAIAMACLVGLMWLSYFIASFRLFARTRSMWSFNPETNILLNVPLHGTILTRPLLESELVI
GAVILRGHLRIAGHHLGRCDIKDLPKEITV**ATSRTLSYY**KLGASQRVAGDSGFAAYSRYRIGNYKLNTDH
SSSSDNIALLVQ

B) **N protein**

MSDNGPQNQRNAPRITFGGPSDSTGSNQNGERSGARSKQRRPQGLPNNTASWFTALTQHGKEDLKFPRGQ
GVPINTNSSPDDQIGYYRRATRRIRGGDGKMKDLSPRWYFYYLGTGPEAGLPYGANKDGIIWVATEGALN
TPKDHIGTRNPANNAAIVLQLPQGTTLPKGFYAEGSRGGSQASSRSSSRSRNSSRNSTPGSSRGTSPARM
AGNGGDAALALLLLDRLNQLESKMSGKGQQQQGQTVTKKSAAEASKKPRQKRTAT**KAYNVTQAFGRRGPE**
QTQGNFGDQELIRQGTDYKHWPQIAQFAPSASAFFGMSRIGMEVTPSGTWLTYTGAIKLDDKDPNFKDQV
ILLNKHIDAYKTFPPTEPKKDKKKKADETQALPQRQKKQQTVTLLPAADLDDFSKQLQQSMSSADSTQA

Figure S3. COVID-19 membrane protein, M (A) and nucleocapsid protein, N (B) epitopes included in the genetic constructs.

A. The epitope ATSRTLSYY (aa 171-179) of the M protein that was included in the genetic constructs 3 and 4 is reported in blue.

B. The long peptide of the N protein comprising the 3 epitopes (highlighted in yellow) KAYNVTQAF (aa 266-274), ELIRQGTDY (aa 290-298) and GMEVTPSGTWLTYTGAIKLD (aa 321-340) that was included in the genetic constructs 3 and 4 is reported in violet.

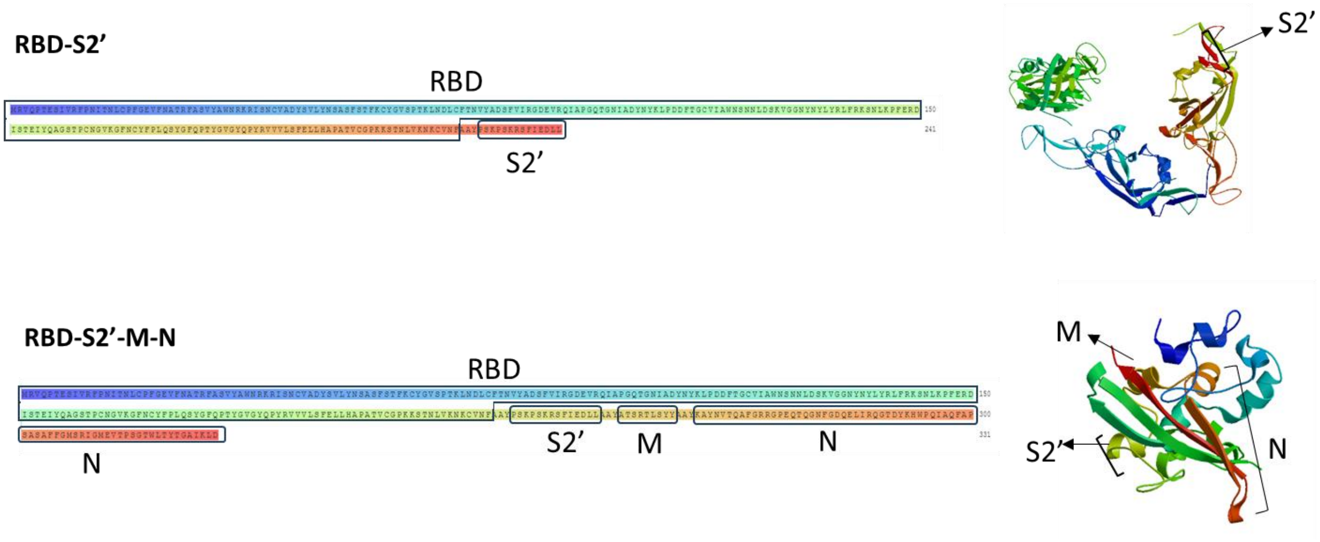

Figure S4. Tri-dimensional prediction of the designed chimeric proteins.

The aminoacidic sequence and 3D model of the constructs RBD-S2' and RBD-S2'-M-N are shown. The 3D model was obtained with the Protean 3D software (Lasergene, DNA star).

The S2’ epitope in the 3D model of the chimeric protein RBD-S2’ is in red.

The S2’ epitope in the 3D model of the chimeric protein RBD-S2’-M-N is in yellow, the M epitope in light brown, the N epitope from orange to red.

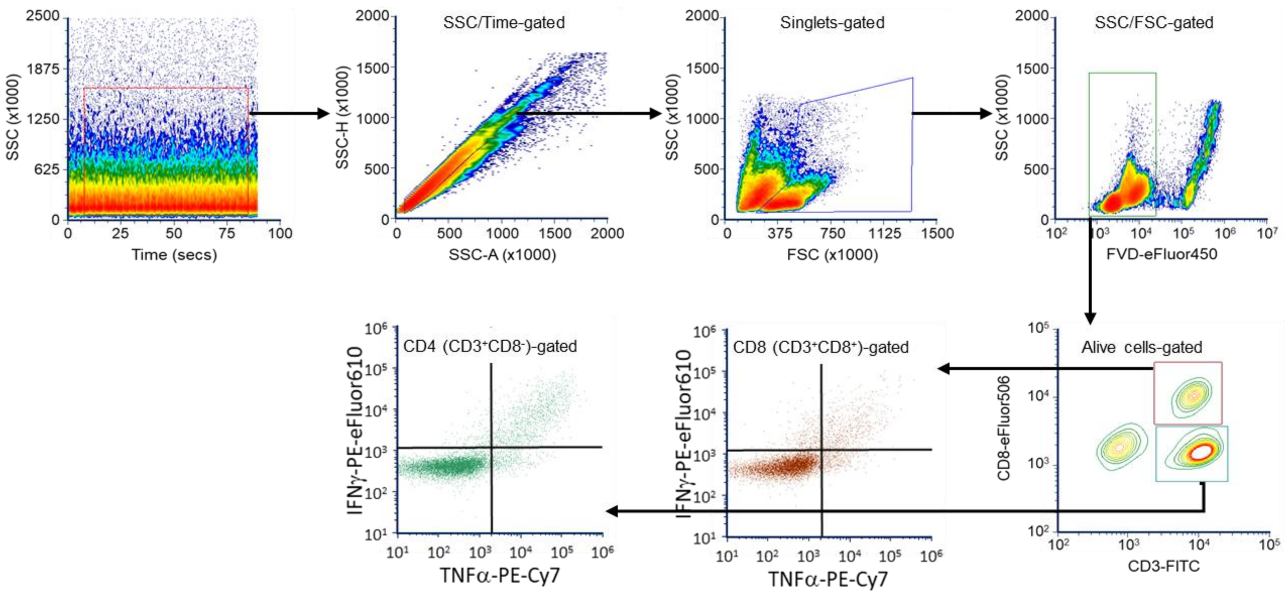

Figure S5. Gating strategy for the identification of cytokine-secreting antigen-specific T cells by flow cytometry

After removal of red blood cells and dissociation to single cell suspension, spleen cells were cultured with medium alone or stimulated with either RBD-S2'-M-N or RBD or N protein for 5 days. During the last 5 hours of culture, cytokine production was boosted by adding again the protein, in the presence of inhibitors of the intracellular secretory/transport pathways (monensin and brefeldin A). Parallel cultures received medium (negative controls) or PMA/ionomycin (positive controls).

At the end of the re-stimulation period, cells were collected and analyzed by flow cytometry using a 5 colors panel designed and verified in preliminary experiments. A hierarchical gating strategy was used to ensure quality of data in each single mouse sample for each of the culture conditions. First, events were plotted against time of acquisition to assess the stability of the conditions. Then, an SSC-H vs. SSC-A plot on Time-gated events was used to select single events (cells) and rule out doublets. SSC vs. FSC plot on single events was used to select relevant cells according to their dimensions and complexity, excluding fragments and debris. Alive cells were identified as cells negative to the staining with Fixable Viability Dye (FVD)-eFluor450 in side scatter/forward scatter (SSC/FSC)-gated events. CD3 and/or CD8 expression was analyzed in FVD negative cells to identify CD4 ($CD3^+CD8^-$) and CD8 ($CD3^+CD8^+$) T cells. Finally, IFN-γ and TNF-α expression were assessed in CD4- and CD8-gated cells. Quadrants in CD4- and CD8-gated plots used to define positive cells according to IFN-γ-PE-eFluor610 and TNF-α-PE-Cy7 fluorescence intensities were set using Fluorescence Minus One (FMO) controls and cells cultured with medium alone.
